# Death in 12–24-Year-Old Youth in Nova Scotia: High Risk of Preventable Deaths for Males, Socially Deprived and Rural Populations—A Report from the NSYOUTHS Program

**DOI:** 10.1155/2010/769075

**Published:** 2010-07-28

**Authors:** T. J. B. Dummer, S. Bellemare, N. MacDonald, L. Parker

**Affiliations:** ^1^Population Cancer Research Program, Dalhousie University, 1494 Carlton Street, Halifax, NS, Canada B3H 3B7; ^2^Children's Hospital of Eastern Ontario, 401 Smyth Road, Ottawa, ON, Canada K1H 8L1; ^3^Division Pediatric Infectious Diseases, Dalhousie University, IWK Health Centre/Canadian Centre for Vaccinology, 5850-5980 University Avenue, P.O. Box 9700, Halifax, NS, Canada B3K 6R8

## Abstract

Deaths from avoidable causes represent the largest component of deaths in young people in Canada and have a considerable social cost in relation to years of potential life lost. We evaluated social and demographic determinants of deaths in youth aged 12–24 years in Nova Scotia for the period 1995–2004. Youth most at risk of death were males, the more socially deprived, and those living in rural areas. There was a five-fold increase in suicides and a three-fold increase in injury deaths in males compared to females and a substantial component of these deaths were amongst males living in rural areas. Initiatives and prevention policies should be targeted towards specific at-risk groups, particularly males living in rural areas. Published vital statistics hide these important trends and thus provide only limited evidence with which to base-prevention initiatives.

## 1. Introduction

Youth is the developmental period encompassing the ages of 12 to 24 years. This is a period characterized by significant physical and emotional maturation during which individuals undergo many developmental changes as they mature into adults. Youth are neither children nor small adults and because there is no easily recognisable age standard to define this transition period, youth health needs often remain hidden by the use of routinely available data [1]. The presentation of health statistics by broad age categorisations can hide important trends associated with the dynamic and changeable nature of youth health. Our definition of youth includes ages at the upper and lower extremes of the youth age range. For the purpose of presenting health statistics, individuals at these ages are often subsumed into broad age categories and consequently defined as either children or adults. By presenting data disaggregated by age we avoid the issues associated with these broad definitions. 

The NSYOUTHS (Nova Scotia Youth Outcome and Utilization of the Health System) database provides an evidence base for understanding the complex interactions that determine youth health and wellbeing. It is a multifaceted population study examining multiple aspects of youth health experience and health care, including health service utilization, transition from pediatric to adult care, and variations in mortality. Understanding the unique health needs and experiences of youth and the factors that affect their health has the potential to lead to significant innovations in the planning and delivery of health and other services to this population. Research findings from the NSYOUTHS project will support the development and evaluation of intra- and intersectoral programs and policies for youth health.

Mortality amongst youth in Canada has declined substantially since the 1970s, although the decrease over the last decade has been less marked [2, 3]. Deaths from avoidable causes—injuries and suicides—remain the largest component of youth deaths and present a significant social problem in terms of years of life lost [4, 5]. This paper evaluates socioeconomic, demographic, and geographic trends in youth mortality in Nova Scotia, 1995–2004—focussing specifically on deaths from avoidable causes including injury and suicide. Whilst data from Canadian Vital Statistics and other national datasets are available describing broad trends in death rates in youth there is a paucity of information on youth deaths disaggregated by age, residential location, or socioeconomic status. The lack of disaggregated data on deaths within the broad period defined as youth limits our ability to define appropriate health and social care activities for this important cohort. The aim of this paper is to fill this clear gap in the evidence required to plan effectively youth death prevention activities. 

## 2. Materials and Methods

### 2.1. Data Sources, Data Capture, and Record Linkage

The NSYOUTHS database is a comprehensive longitudinal dataset comprising health and health care utilisation information for all youth aged 12 to 24 years resident in Nova Scotia between 1995 and 2004 and eligible for insured health care services paid for by Nova Scotia Medical Services Insurance (MSI). We used a modified version of the World Health Organisation definition of youth [6]. The database was created by linking five administrative health databases: Nova Scotia Patient Registry, MSI Physician Billings, Canadian Institute of Health Information (CIHI), Hospital Discharge Abstract Database (DAD), Mental Health Outpatient Information System, and Vital Statistics Death Registry. All provincial residents are eligible for health services covered by MSI, only those paid for privately were not captured in the database. Members of the armed forces and Royal Canadian Mounted Police do not have MSI Health Cards and were excluded from the dataset. The demographic and health variables provided from each dataset are listed in Table 1. 

In order to maintain patient anonymity and confidentiality all MSI health card numbers were encrypted. Individual databases were linked by the Population Health Research Unit (PHRU) at Dalhousie University using the encrypted MSI health card number. 

**Figure 1:**
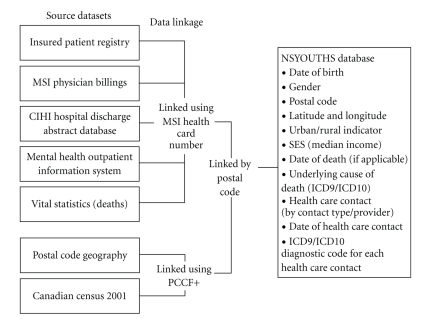
Overview of the NSYOUTHS database.

Additional demographic variables were extracted from the 2001 Canadian Census and the postal code geography database held by PHRU. Median household income was derived from the 2001 census of population [7] (dissemination area statistics) and linked to residential postal codes via the Postal Code Conversion File (PCCF+) [8]. Median household income was grouped into quartiles and linked to individuals via their home postal code, providing an indicator of community socioeconomic status (SES) for individuals. Urban/rural residential status, as defined in the PCCF+, was assigned to individuals using their residential postal code. Finally, a grid reference (latitude and longitude) was assigned to each individual postal code using the PCCF+. Figure 1 provides an overview of the databases and record linkage processes used to create the NSYOUTHS database. 

### 2.2. Analytical Methods

Mortality rates—over time and by age, gender, urban/rural residence, and SES—were calculated per 100,000 youth person years. 95% confidence intervals (CIs) were derived using the Poisson distribution [9]. Underlying cause of death was grouped into avoidable deaths (categorised into self-harm/suicide or injury) and all other disease-related deaths. Poisson regression was used to estimate incidence-rate ratios (IRRs) and 95% CIs adjusted for gender, age, year, urban/rural residence, and SES. Statistical analysis was undertaken using SAS 9.1 [10]. 

The location of residence of all individuals in the NSYOUTHS database were captured into a Geographical Information System (GIS) [11] using the latitude and longitude of their home postal code. Individuals were assigned to a Nova Scotia community health board and mortality rate maps were produced. The observed and expected number of deaths, by urban/rural location and categories of socioeconomic status, was compared, where the expected number of deaths was estimated using Poisson regression, adjusting for gender, age, and time period. 

### 2.3. Ethics

The project received ethics approval from the IWK Health Centre Research Ethics Board and approval from the PHRU Access to Data and Information Management Committee.

## 3. Results

The NSYOUTHS database is a cohort of 314,983 individual youth (158,179 males and 156,804 females), representing over 1.6 million person years during the 10 year study period. During each year of the study there were approximately 170,000 individuals in the database. There were 718 deaths in the cohort, an overall death rate of 43 (95% CI 40–46) per 100,000 youth. Of these deaths, 55 (8%) did not have an underlying cause of death recorded, primarily because health card number, which was used for record linkage, is not a requirement on death registrations provided by vital statistics and is missing in some cases. 

**Figure 2:**
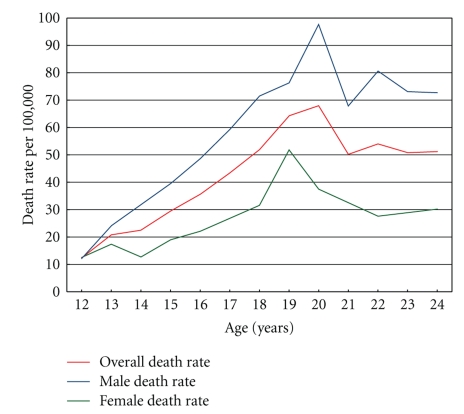
Death rates by age per 100,000 youth, overall and by male and female.

Death rates, overall and stratified by gender, are presented in Table 2. During the study period, there was no significant change in death rates over time. There was a two-fold increase in male compared to female death rates; male rate 58 (95% CI: 53–63) per 100,000 youth, female rate 27 (95% CI: 24–31) per 100,000 youth, adjusted IRR 2.1 (95% CI 1.8–2.5). Death rates were markedly higher in rural compared to urban areas; rural death rate 51 (95% CI: 46–56) per 100,000, urban death rate 38 (95% CI: 34–41) per 100,000, adjusted IRR 1.3 (95% CI: 1.1–1.5) in rural compared to urban areas. There was a trend for higher death rates in the lower socioeconomic groups, adjusted IRR was 1.6 (95% CI: 1.3–2.0) comparing 1st (lowest) to 4th (highest) SES quartile. Death rates by age increased steadily from the lowest rate at age 12 years, peaking at age 20 years before declining at age 21, although the rate at age 24 was very similar to the rate at ages 22 and 23 years (see Table 2 and Figure 2). The male death rate was consistently higher than the female rate and although the overall trend by age was similar the gap between male and female rates widened with older age.

Because of the markedly increased risk of death for males compared to females the Poisson regression analysis was stratified by gender (see Table 2). An increased risk of death in rural areas compared to urban areas was apparent for both males and females, although this risk was significant only for males. The trend for risk of death by year of age was markedly different for males compared to females, reflecting the large increase in the number of male compared to female deaths. For example the highest IRR (compared with age 12) for females was 4.4 (95% CI 1.9–10) at age 19, whereas for males it was 9.0 (95% CI 4.1–19.7) at age 20. There was no significant difference in the trend in risk of death by socioeconomic status or time period for males or females. 

A total of 663 deaths in the cohort had a cause of death coded, of these 33% were disease-related with the remaining 67% due to causes defined as avoidable (51% from injury and 16% from suicide/self-harm). Death rates by cause per 100,000 person years were: disease-related 13 (95% CI 11–15); injury 20.0 (95% CI 18–22); and suicide/self-harm 6.3 (95% CI 5.2–7.6), see Table 3. There was a statistically significant three-fold higher rate of deaths from injury in males (adjusted IRR 2.8, 95% CI 2.2–3.6) and a five-fold higher rate of deaths from suicides in males (adjusted IRR 5.4, 95% CI: 3.1–9.4) compared to females. By contrast, the death rates for disease-related deaths for males and females were almost identical. A small proportion of deaths (*n* = 20) categorised as unintentional injury were from causes related to interpersonal violence (homicide/assault) (results not reported in the Table 3). The interpersonal violence death rate was higher for males (IRR 1.8, 95% CI: 0.7–4.5), lower in rural compared to urban areas (IRR 0.4, 95% CI: 0.2–1.2) and higher in the lower socioeconomic groups (IRR 2.0, 95% CI 0.7–5.9 comparing the highest to lowest socioeconomic quartile). However, the 95% CIs indicated none of these findings were statistically significant. 

**Figure 3:**
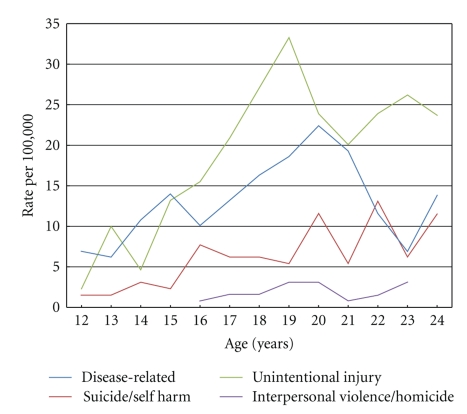
Death rates by age per 100,000 youth by cause category (disease-related, injury, interpersonal violence, suicide/self harm).

There was significantly higher rate of deaths from injury in rural areas compared to urban areas (IRR 1.7, 95% CI: 1.4–2.2), whereas deaths from suicides were just marginally higher in rural areas and deaths from disease-related causes were the same in rural and urban areas. In all three cause groups death rates were higher in the lowest socioeconomic category compared to the most affluent category—IRR comparing lowest to highest socioeconomic quartile: injury deaths 1.6 (95% CI: 1.2–2.3), self-harm/suicide deaths 3.0 (95% CI: 1.5–6.2), and disease-related deaths 1.6 (95% CI: 1.1–2.5). Deaths from injury displayed the largest variation by year of age compared to the other cause categories. Over time, disease-related death rates changed a little, and there was a slight decline in suicide death rates. However, there was a marked, but statistically nonsignificant, higher death rate from injury in 2004 compared to earlier time periods, although in general death rates from injury also declined over time. There was significant variation in risk of death from suicide and injury by year of age, compared to very little variation in disease-related deaths by age. The highest risk of death from injury was at age 19 (adjusted IRR 23.2, 95% CI 5.6–95.5 compared to age 12) although the risk for subsequent ages did not decline substantially. The highest risk for suicide was at age 22 (adjusted IRR 8.1, 95% CI 1.9–35.4 compared to age 12). Although there was variation in death rates from each cause group over time this temporal fluctuation was nonsignificant (Table 3). Figure 3 graphically illustrates death rates by cause (disease-related, injury, suicide/self-harm and interpersonal violence/homicide) by year of age, highlighting the peak in injury deaths at age 19, suicides at age 22, and the relatively small rate of deaths from interpersonal violence/homicide in this cohort. 

**Figure 4:**
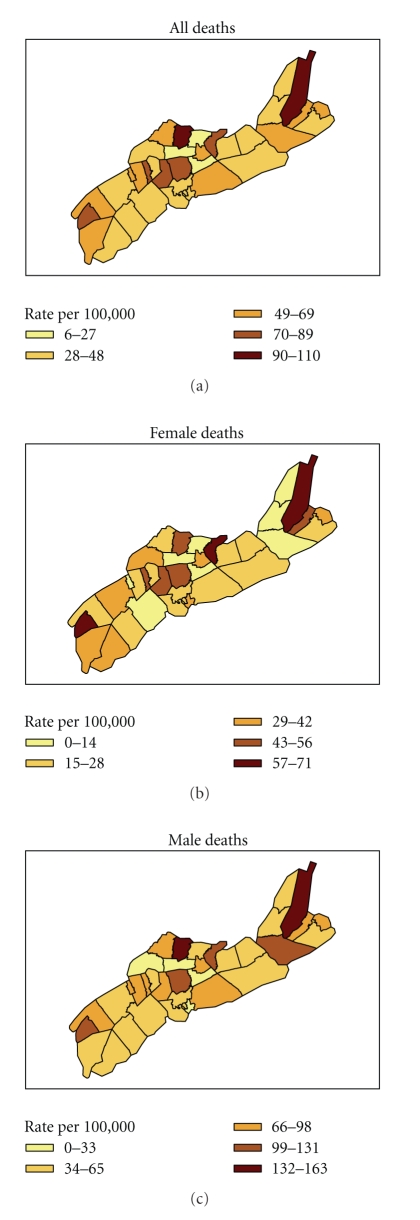
Death rates per 100,000 youth by Community Health Board.

There was substantial geographic variation in death rates, see Figure 4 for rates by community heath boards. In rural areas, an excess of 55 deaths was observed compared to the number expected assuming no extra-Poisson spatial variation in death rates, whereas in urban areas there were 34 less deaths than expected. In the most deprived category of socioeconomic status, there were 31 more deaths than expected as predicted by the Poisson regression model, whereas in the most affluent socioeconomic category 41 less deaths than expected were observed. 

## 4. Discussion

Youth deaths in Nova Scotia were twice as high in males compared to females and significantly higher in rural compared with urban areas, which was partly due to a 40% higher rate of death in males in rural areas. Death rates in the most deprived quintile of socioeconomic status were increased by 50% and 80% for males and females, respectively. The substantial difference in male compared to female deaths was due to large differences in deaths from avoidable causes—suicides were 5.4 times higher and injury deaths were 2.8 times higher for males compared to females. 

The Nova Scotia youth death rate during the study period was slightly lower than the 2000–2004 Canadian rate of 47 deaths per 100,000 (age 15–19 years) and 59 deaths per 100,000 (age 20–24 years) [3], similar to the UK rate of 36 per 100,000 (age 15–19 years) and 50 per 100,000 (age 20–24 years) for the same time period [12], and considerably lower than the US rate of 80 per 100,000 for ages 15–24 years, 1999–2004 [13]. The Nova Scotia male youth death rate was lower than the Australian rate of 63 per 100,000 in 2002, and very similar to the Australian female rate of 25 per 100,000 [14]. Similar to the Canadian national rate the Nova Scotia male death rate was double the female death rate, whereas in the US, UK, and Australia it around 2.5 times higher [3, 12, 13]. 

Injury deaths in Nova Scotia were generally lower than those reported for both Canada and the US: Canada rate 22 per 100,000 for ages 15–19 years and 24 per 100,000 for ages 20–24 years, US rate 36 per 100,000 for youth aged 15–24 for the period 1999–2004. The proportion of deaths from external causes (injury and suicide) was similar in Nova Scotia (63%) and Australia (70%) [3, 13]. 

Historically, the Canadian youth suicide rate has been higher than in the US, Australia and the UK [4]. However, the Nova Scotia youth suicide rate was considerably lower than the Canadian rate of 10 per 100,000 (ages 15–19 years) and 14 per 100,000 (ages 20–24 years) and the US rate of 10 per 100,000 for 15–24 year olds. Although Nova Scotia has the lowest rate of youth suicides in Canada, the five-fold difference between male and female suicide rates was higher than the Canadian average and similar to US data [4]. 

The geographic heterogeneity in youth deaths in this study reflects partly the substantial and significant variation in deaths from avoidable causes between rural and urban areas. However, some of the spatial variation in death rates may be due to variation in socioeconomic risk factors, which were defined at the community level using postal code data and it is known that socioeconomic status varies between geographic communities in Nova Scotia. 

The NSYOUTHS database is a comprehensive longitudinal resource which enabled us to explore temporal trends in death rates by such factors as age, gender, socioeconomic status, and urban-rural residence—analyses that are not possible using published vital statistics, either Canadian or international. Whilst it is well known that the two major causes of death in youth are injuries and suicides, and that the burden of these deaths is predominantly in males, published vital statistics do not identify socioeconomic or urban/rural trends.

This study highlighted the important role living in a rural area has on youth suicides and injury deaths and has therefore identified a focus for prevention initiatives. Further, we were able to present death rates by year of age, as opposed to more typical analyses by age categories. This allowed us to highlight that the injury death rate for older youth did not decline substantially after the peak rate at age 19. National and provincial statistics are commonly reported by broad age groups, thus masking important variations within these groups and, again, providing only limited evidence on which to base prevention strategies. 

The cohort was established using the provincial MSI patient registry to create the relevant age-defined study population, ensuring the calculation of accurate person years at risk. By combining provincial MSI and CIHI DAD data with Vital Statistics data to identify deaths within our cohort, we were able to ensure that ascertainment of deaths was as complete as possible. However, a limitation of the database is that around 7% of the deaths do not have a cause assigned because, in these cases, the vital statistics death registration did not have a health card number assigned and record linkage was not possible. 

## 5. Conclusion

We found significant inequality in death rates in Nova Scotia youth and individuals most at risk of death were males, the more socially deprived and those living in rural areas. A large component of this increased risk was due to high death rates from injury and suicide amongst males, especially males living in rural areas. Whilst these injury deaths declined after a peak at age 19 years, the rates remained substantial through to age 24. Thus, initiatives to prevent youth deaths must be both sex- and age-specific. Deaths from suicide and injury are largely preventable through medical, social care, and public health initiatives. The development of appropriate mental health, educational, road safety, and transport planning policies are essential to deal with these avoidable deaths. This study shows that, in addition to developing suitable prevention strategies, all initiatives and policies should be targeted towards specific at-risk groups, particularly males living in rural areas. 

## Figures and Tables

**Table 1:** Variables and health data sources.

Database	Variable
Nova Scotia Patient Registry	MSI Health card number
	Date of birth
	Gender
	Postal code of residence

Medical Services Insurance (MSI) Physician Billings	MSI Health card number
	Health care contact location
	Date of consultation
	Diagnostic code (ICD9 1995–2000/ICD10 2001–04)

Canadian Institute of Health Information (CIHI) Hospital Discharge Abstract Database (DAD)	MSI Health card number
	Health care contact location
	Date of admission
	Diagnostic code (ICD9 1995–2000/ICD10 2001–04)

Mental Health Outpatient Information System	MSI Health card number
	Health care contact location
	Date of consultation
	Diagnostic code (ICD9 1995–2000/ICD10 2001–04)

Vital Statistics Death Registry	Date of death
	Place of death
	Underlying cause of death (ICD9 1995–99/ICD10 2000–04)

**Table 2:** Death rates (per 100,000 youth) and incident-rate ratios (IRR), overall and by gender.

	Death Rates						Incident Rate Ratios (IRR)							
Variable	Overall		Male		Female		Unadjusted		Adjusted		Males		Females	
	Rate	95% CI	Rate	95% CI	Rate	95% CI	IRR	95% CI	IRR	95% CI	IRR	95% CI	IRR	95% CI
Gender														
Female	27.0	23.6–30.8	—	—	—	—	1.0	—	1.0	—	—	—	—	—
Male	57.9	52.9–63.3	—	—	—	—	2.1	1.8–2.5	2.2	1.8–2.5	—	—	—	—

Urbanicity														
Rural	50.6	45.5–56.1	70.6	62.3–79.7	29.3	23.9–35.6	1.4	1.2–1.6	1.3	1.1–1.5	1.4	1.1–1.7	1.2	0.9–1.6
Urban	37.5	33.5–41.7	49.4	43.1–56.4	25.6	21.1–30.8	1.0	—	1.0	—	1.0	—	1.0	—

SES														
1 (low)	50.4	43.6–58.0	65.5	54.6–78.0	35.3	27.4–44.7	1.7	1.3–2.1	1.6	1.3–2.0	1.5	1.2–2.0	1.8	1.2–2.6
2	45.5	39.0–52.7	66.8	55.8–79.2	23.6	17.2–31.6	1.6	1.3–2.0	1.4	1.1–1.8	1.5	1.1–2.0	1.2	0.8–1.9
3	47.8	41.1–55.2	63.7	53.1–75.9	31.2	23.7–40.2	1.6	1.3–2.0	1.5	1.1–1.8	1.5	1.1–1.9	1.4	0.9–2.2
4 (high)	29.7	24.5–35.6	39.7	31.4–49.5	19.3	13.6–26.7	1.0	—	1.0	—	1.0	—	1.0	—

Age at death														
12	12.3	7.0–20.0	12.2	5.2–23.7	12.6	5.5–24.9	1.0	—	1.0	—	1.0	—	1.0	—
13	20.8	13.7–30.3	24.1	13.8–39.1	17.4	8.7–31.1	1.7	0.9–3.1	1.8	0.9–3.4	2.1	0.9–5.3	1.4	0.5–3.7
14	22.5	15.0–32.2	31.8	19.7–48.7	12.7	5.5–25.0	1.8	1.0–3.4	2.0	1.1–3.8	2.9	1.2–6.8	1.1	0.4–3.1
15	29.5	20.9–40.4	39.5	25.8–57.8	19.0	9.8–33.2	2.4	1.3–4.3	2.5	1.4–4.7	3.4	1.5–8.0	1.6	0.6–4.0
16	35.6	26.1–47.5	48.6	33.3–68.6	22.1	12.1–37.1	2.9	1.6–5.1	3.3	1.8–6.0	4.6	2.0–10.4	2.0	0.8–4.9
17	43.4	32.8–56.4	59.3	42.2–81.1	26.9	15.7–43.0	3.5	2.0–6.2	3.7	2.0–6.6	5.2	2.3–11.6	2.1	0.9–5.2
18	51.9	40.2–65.9	71.5	52.6– 95.1	31.6	19.3–48.8	4.2	2.4–7.3	4.5	2.5–8.1	6.2	2.8–13.7	2.9	1.2–6.7
19	64.3	51.2–79.7	76.3	56.6–100.5	51.9	35.7–72.9	5.2	3.1–8.9	5.7	3.2–10.0	6.9	3.1–15.3	4.4	1.9–10.0
20	68.0	54.5–83.7	97.8	75.3–124.9	37.5	24.0–55.7	5.5	3.2–9.4	6.0	3.4–10.5	9.0	4.1–19.7	3.0	1.3–7.0
21	50.2	38.8–64.0	67.8	49.3–91.1	32.6	20.2–49.8	4.1	2.4–7.1	4.5	2.5–8.0	6.2	2.8–13.8	2.7	1.1–6.4
22	54.0	42.1–68.2	80.6	60.2–105.7	27.6	16.4–43.7	4.4	2.6–7.5	4.7	2.6–8.3	7.1	3.2–15.6	2.3	0.9–5.5
23	50.8	39.3–64.6	73.1	53.7–97.2	28.9	17.4–45.2	4.1	2.4–7.1	4.5	2.5–8.0	6.2	2.8–13.8	2.7	1.1–6.4
24	51.2	39.7–65.0	72.7	53.4–96.7	30.2	18.4–46.6	4.2	2.4–7.2	4.7	2.6–8.3	6.8	3.1–15.1	2.5	1.1–6.1

Year of death														
1995	42.2	33.0–53.2	58.2	43.2–76.7	26.0	16.3–39.4	1.0	—	1.0	—	1.0	—	1.0	—
1996	44.1	34.7–55.3	58.2	43.2–76.8	29.7	19.2–43.9	1.1	0.8–1.4	1.0	0.7–1.4	0.9	0.6–1.4	1.1	0.6–2.0
1997	47.4	37.6–59.0	64.4	48.5–83.9	30.0	19.4–44.3	1.1	0.8–1.5	1.0	0.7–1.4	1.0	0.7–1.5	1.1	0.6–2.0
1998	51.3	41.0–63.3	76.6	59.1–97.7	25.3	15.7–38.7	1.2	0.9–1.7	1.1	0.8–1.5	1.2	0.8–1.8	0.9	0.5–1.6
1999	38.8	29.9–49.4	49.5	35.7–66.9	27.8	17.6–41.7	0.9	0.7–1.3	0.8	0.6–1.2	0.8	0.5–1.2	1.0	0.5–1.8
2000	49.6	39.5–61.5	69.7	53.1–90.0	29.0	18.6–43.2	1.2	0.9–1.6	1.0	0.7–1.4	1.1	0.7–1.6	1.0	0.5–1.9
2001	38.3	29.5–48.9	54.5	39.9–72.7	21.8	12.9–34.5	0.9	0.6–1.3	0.8	0.6–1.1	0.8	0.5–1.3	0.7	0.4–1.4
2002	33.4	25.2–43.4	43.7	30.7–60.2	22.9	13.8–35.8	0.8	0.6–1.1	0.7	0.5–1.0	0.6	0.4–1.0	0.8	0.4–1.5
2003	39.2	30.3–49.9	49.3	35.5–66.6	28.9	18.5–43.0	0.9	0.7–1.3	0.8	0.6–1.2	0.8	0.5–1.2	1.1	0.6–1.9
2004	42.0	32.8–53.0	54.9	40.3–73.0	28.8	18.5–42.9	1.0	0.7–1.4	0.9	0.6–1.3	0.8	0.5–1.2	1.2	0.6–2.1

**Table 3:** Death rates (per 100,000 youth) and adjusted Incidence-Rate Ratios (IRR) by cause group: avoidable deaths and other deaths (disease-related).

Variable	Death Rates						Incident Rate Ratios (IRR)					
	Avoidale deaths						Avoidable deaths					
	Injury		Self harm/suicide		Disease related		Injury		Self harm/suicide		Disease related	
	Rate	95% CI	Rate	95% CI	Rate	95% CI	IRR	95% CI	IRR	95% CI	IRR	95% CI
Gender												
Female	10.3	8.3–12.8	1.9	1.1–3.1	12.4	10.1–15.0	1.0	—	1.0	—	1.0	—
Male	29.5	25.9–33.4	10.6	8.5–13.0	13.7	11.4–16.5	2.8	2.2–3.6	5.4	3.1–9.4	1.1	0.8–1.5

Urbanicity												
Rural	26.9	23.2–31.0	7.9	5.9–10.2	13.0	10.5–16.0	1.7	1.4–2.2	1.2	0.8–1.8	0.9	0.7–1.3
Urban	15.3	12.9–18.1	5.4	4.0–7.2	13.6	11.3–16.3	1.0	—	1.0	—	1.0	—

SES												
1 (low)	24.1	19.4–29.5	8.3	5.7–11.7	16.0	12.3–20.6	1.6	1.2–2.3	3.0	1.5–6.2	1.6	1.1–2.5
2	21.7	17.3–26.9	8.5	5.9–12.0	12.4	9.1–16.4	1.3	0.9–1.8	3.2	1.6–6.5	1.4	0.9–2.1
3	22.5	18.0–27.7	5.9	3.8–8.9	16.0	12.3–20.5	1.4	1.0–2.0	1.9	0.9–4.1	1.6	1.1–2.4
4 (high)	14.2	10.7–18.5	2.6	1.2–4.7	9.0	6.3–12.6	1.0	—	1.0	—	1.0	—

Age at death												
12	2.3	0.5–6.7	1.5	0.2–5.6	6.9	3.2–13.1	1.0	—	1.0	—	1.0	—
13	10.0	5.3–17.1	1.5	0.2–5.6	6.2	2.7–12.2	6.0	1.3–26.7	1.0	0.1–7.1	0.8	0.3–2.1
14	4.6	1.7–10.1	3.1	0.8–7.9	10.8	5.9–18.2	3.0	0.6–14.8	2.0	0.4–11.0	1.4	0.6–3.4
15	13.2	7.7–21.1	2.3	0.5–6.8	14.0	8.3–22.1	8.5	2.0–36.7	1.0	0.1–7.1	1.8	0.8–4.0
16	16.3	10.1–24.9	7.7	3.7–14.2	10.1	5.4–17.2	10.5	2.5–44.7	5.0	1.1–22.9	1.4	0.6–3.4
17	22.5	15.1–32.3	6.2	2.7–12.2	13.2	7.7–21.1	13.0	3.1–54.8	4.0	0.9–18.8	1.7	0.7–3.8
18	28.7	20.2–39.5	6.2	2.7–12.2	16.3	10.1–24.9	17.0	4.1–70.9	4.0	0.9–18.8	2.2	1.0–4.9
19	36.4	26.7–48.4	5.4	2.2–11.2	18.6	11.9–27.6	23.2	5.6–95.5	3.5	0.7–16.8	2.6	1.2–5.5
20	27.0	18.8–37.6	11.6	6.5–19.1	22.4	15.0–32.2	17.2	4.1–71.7	7.5	1.7–32.9	3.0	1.4–6.4
21	20.9	13.8–30.3	5.4	2.2–11.1	19.3	12.5–28.5	13.4	3.2–56.5	2.5	0.5–13.0	2.7	1.3–5.8
22	25.4	17.5–35.7	13.1	7.6–21.0	11.6	6.5–19.1	15.7	3.8–65.8	8.1	1.9–35.4	1.6	0.7–3.6
23	29.2	20.7–40.1	6.2	2.7–12.1	6.9	3.2–13.1	18.0	4.3–74.9	3.6	0.7–17.2	1.1	0.4–2.5
24	23.7	16.1–33.6	11.5	6.4–18.9	13.8	8.1–21.7	16.4	3.9–68.4	6.1	1.4–27.2	1.9	0.8–4.2

Year of death												
1995	20.5	14.3–28.6	5.9	2.8–10.8	8.8	4.9–14.5	1.0	—	1.0	—	1.0	—
1996	20.0	13.9–28.0	5.9	2.8–10.8	14.7	9.5–21.7	0.9	0.6–1.5	0.8	0.3–1.9	1.4	0.7–2.6
1997	23.1	16.4–31.6	7.7	4.1–13.2	11.9	7.2–18.3	1.0	0.6–1.6	1.0	0.5–2.4	1.1	0.6–2.2
1998	19.1	13.0–26.9	10.7	6.4–17.0	18.5	12.6–26.2	0.9	0.5–1.4	1.5	0.7–3.2	1.7	0.9–3.1
1999	19.1	13.1–27.0	6.0	2.9–11.0	8.4	4.6–14.0	0.8	0.5–1.4	0.9	0.4–2.1	0.8	0.4–1.6
2000	21.5	15.1–29.8	5.4	2.5–10.2	19.1	13.1–27.0	1.0	0.6–1.5	0.6	0.2–1.6	1.6	0.9–3.0
2001	16.8	11.1–24.2	5.4	2.5–10.2	14.4	9.2–21.4	0.7	0.4–1.2	0.6	0.2–1.6	1.2	0.6–2.4
2002	17.9	12.1–25.5	4.2	1.7–8.6	10.1	5.9–16.2	0.7	0.4–1.2	0.6	0.2–1.6	0.8	0.4–1.7
2003	15.4	10.1–22.6	8.3	4.5–14.0	13.7	8.7–20.5	0.7	0.4–1.2	1.1	0.5–2.6	1.2	0.6–2.3
2004	26.6	19.4–35.6	3.6	1.3–7.7	11.2	6.8–17.6	1.1	0.7–1.8	0.6	0.2–1.5	1.0	0.5–2.0
